# Heterogeneous indications and the need for viability assessment: An international survey on the use of machine perfusion in liver transplantation

**DOI:** 10.1111/aor.14061

**Published:** 2021-09-08

**Authors:** Damiano Patrono, Davide Cussa, Federica Rigo, Renato Romagnoli, the Liver Machine Perfusion Survey Group, Roberta Angelico, Maria Irene Bellini, Eliano Bonaccorsi‐Riani, Isabel M. A. Brüggenwirth, Zoltan Czigany, Riccardo De Carlis, Vincent E. De Meijer, Daniele Dondossola, Dilmurodjon Eshmuminov, Davide Ghinolfi, Amelia J. Hessheimer, Dagmar Kollmann, Quirino Lai, Georg Lurje, Tommaso M. Manzia, Arianeb Merhabi, Fabio Melandro, David Nasralla, Arash Nickkholgh, Duilio Pagano, Michel Rayar, Maria Cristina Saffioti, Annemarie Weissenbacher, Alfonso W. Avolio, Paolo De Simone, Costantino Fondevila, Wayel Jassem, Malcolm Macconmara, Robert J. Porte, Matteo Ravaioli, Markus Selzner, Marco Spada

**Affiliations:** ^1^ General Surgery 2U—Liver Transplant Unit AOU CIttà della Salute e della Scienza di Torino Turin Italy

**Keywords:** ex situ organ perfusion, machine perfusion, normothermic regional perfusion, organ preservation, viability assessment

## Abstract

Although machine perfusion (MP) is being increasingly adopted in liver transplantation, indications, timing, and modality are debated. To investigate current indications for MP a web‐based Google Forms survey was launched in January 2021 and addressed to 127 experts in the field, identified among first and corresponding Authors of MP literature in the last 10 years. The survey presented 10 real‐life cases of donor–recipient matching, asking whether the liver would be accepted (Q1), whether MP would be used in that particular setting (Q2) and, if so, by which MP modality (Q3) and at what timing during preservation (Q4). Respondents could also comment on each case. The agreement was evaluated using Krippendorff's alpha coefficient. Answers from 39 (30.1%) participants disclosed significant heterogeneity in graft acceptance, MP indications, technique, and timing. Agreement between respondents was generally poor (Q1, *α* = 0.11; Q2, *α* = 0.14; Q3, *α* = 0.12, Q4, *α* = 0.11). Overall, respondents preferred hypothermic MP and an end‐ischemic approach in 56.3% and 81.1% of cases, respectively. A total of 18 (46.2%) participants considered only one MP approach, whereas 17 (43.6%) and 3 (7.7%) considered using alternatively 2 or 3 different techniques. Of 38 comments, 17 (44.7%) were about the use of MP for graft viability assessment before implantation. This survey shows considerable variability in MP indications, emphasizing the need to identify scenarios of optimal utilization for each technique. Viability assessment emerges as a fundamental need of transplant professionals when considering the use of MP.

## Graphical abstract

In this survey, experts in the field of machine perfusion and liver transplantation were confronted to ten real‐world cases of donor‐recipient matching. Respondents were asked whether they would accept the offer and, if so, whether, how and when they would use machine perfusion for each case. The picture, which depicts each respondent answers, highlight significant heterogeneity with regards to graft acceptance and machine perfusion practice.

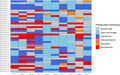

## 1 INTRODUCTION

Machine perfusion (MP) is revolutionizing the field of liver transplantation (LT).^1^, ^2^ The deep interest in MP, a term that encompasses various approaches, is supported by the impelling necessity to increase donor pool and to improve the preservation of grafts from extended criteria donors. This necessity fostered its transition from the experimental setting to the clinical practice. A rapidly growing body of literature supports its use in different scenarios in LT.^3^, ^4^, ^5^, ^6^, ^7^, ^8^, ^9^, ^10^, ^11^, ^12^, ^13^, ^14^, ^15^, ^16^, ^17^, ^18^, ^19^, ^20^, ^21^, ^22^, ^23^ Three randomized controlled trials have been published,^9^, ^11^, ^19^ whereas several others are currently ongoing and results are expected soon.^24^

In keeping with the relatively recent adoption, MP indications and modality appear to be highly variable among different centers. Which grafts should be preserved using MP, in which scenarios are its cost justified, what technique is to be preferred and what is the optimal timing for MP use during preservation are all open questions. Furthermore, the choice of a particular approach may be influenced by each center experience and skills, different organ procurement organizations, and healthcare policies.

Despite its clinical relevance, variability in MP indications among different centers has not been investigated so far. Thus, we sought to explore different approaches to MP in LT by analyzing how some real‐life donor‐recipient scenarios, gathered from the experience of the promoting center, would be managed. This study was inspired by a recent survey from the Zurich group, which evidenced considerable discrepancies in the treatment of colorectal liver metastases even among experts in the field.^25^ The working hypothesis of this survey was that indications for MP are heterogeneous, even among transplant professionals with direct MP experience and a thorough understanding of the advantages and limitations of each technique. This survey aimed to provide an accurate and representative cross‐sectional picture of how different MP modalities are perceived among various groups and regions, to identify the specific areas of interest for future consensus guidelines and highlight the issues and needs of transplant professionals toward MP technology.

## 2 MATERIALS AND METHODS

A web‐based Google Forms survey (https://forms.gle/2DLK3kK1EMCWewbz6) was launched in January 2021 and addressed by email to 127 experts in the field of liver MP, identified by a systematic PubMed search of first and corresponding authors of articles about clinical liver MP in the last 10 years. In case of nonresponse after the first contact, we sent a single reminder to target experts. The survey link was not shared on social media platforms. Geographical location of target experts was as follows: Europe, n = 90 (71%); North America, n = 24 (19%); South America, n = 3 (2%); Asia, n = 10 (8%).

The design and ethics of this survey followed the recommendations “Best Practices for Survey Research” by the American Association for Public Opinion Research (AAPOR) (https://www.aapor.org/). Briefly, respondents were informed about the aim of the survey and that only pooled data would be reported. Anonymity was preserved at each stage. For stratification purposes, geographical location, volume and presence of an established MP program at respondent center was asked in preliminary questions. Noteworthy, also respondents from centers without an established MP program had direct clinical experience with MP and proven knowledge in the field of MP. To safeguard anonymity, email address could be provided on a voluntary basis at a separate link (https://forms.gle/iENmPygSYDrtoEMLA) where respondents could discover how survey cases had actually been managed at the promoting center. The full text of the survey is available as Supporting Information. All participants included as co‐authors accepted to be included and approved the final version of the manuscript. The survey was approved by the local Institutional Review Board that ruled out the necessity for further approval by the local Ethics Committee. Study procedures complied with the 2008 version of the Declaration of Helsinki (https://www.wma.net).

The survey was based on 10 real‐world cases managed at the promoting transplant center in the period 2019‐2020 (Table 1 and Supporting Information). For each case, the most relevant information available during the allocation process was provided. Data included clinical donor data, blood test results, macroscopic evaluation by the retrieving surgeon, availability of a preretrieval biopsy and, if so, histologic features like degree of macrovesicular steatosis. A brief picture of recipient clinical data and degree of urgency was also provided, to allow respondents also answering on the ground of donor–recipient matching.

**TABLE 1:** Brief description of survey cases (for a full description see Supporting Information)

	Donor issues	Recipient issues	MP^1^	Transplanted	Outcome
Case 1	78‐yo DBD with 20% macrosteatosis	None	NMP	Yes	Dead at 6 months due to HCC recurrence; no ITBL
Case 2	63‐yo DBD with 40% macrosteatosis	MELD = 20; BMI = 33	D‐HOPE	Yes	Retransplanted on POD 31st; dead at 3 months due to HHV8 infection
Case 3	49‐yo type II DCD with 35‐minutes asystolic WIT	MELD = 21	NRP + D‐HOPE	Yes	Alive at 12 months; no ITBL
Case 4	56‐yo type III DCD with 49‐minutes fWIT	None	NRP + D‐HOPE	Yes	Alive at 10 months; no ITBL
Case 5	52‐yo DBD with elevated liver enzymes	None	D‐HOPE	Yes	Alive at 14 months; no ITBL
Case 6	42‐yo DBD with steatotic appearance	None	D‐HOPE	Yes	Alive at 14 months; no ITBL
Case 7	76‐yo DBD with elevated liver enzymes	HBV‐related ALF; MELD = 41	NMP	Yes	Alive at 20 months; no ITBL
Case 8	12‐yo DBD with elevated liver enzymes	None	D‐HOPE	Yes	Alive at 21 months; isolated S6 duct ITBL + anastomotic stricture
Case 9	21‐yo type II DCD with 31‐minutes asystolic WIT	None	NRP + NMP	No	Na
Case 10	96‐yo DBD	None	D‐HOPE	Yes	Alive at 2.5 years; no ITBL

Abbreviations: ALF, acute liver failure; BMI, body mass index; DBD, donation after brain death; DCD, donation after circulatory death; D‐HOPE, dual hypothermic oxygenated machine perfusion; fWIT, functional warm ischemia time; HBV, hepatitis B virus; ITBL, ischemic‐type biliary lesion; MELD, model for end‐stage liver disease; MP, machine perfusion; Na, not applicable; NMP, normothermic machine perfusion; NRP, normothermic regional perfusion; WIT, warm ischemia time.

^1^ Choice of machine perfusion technique at the promoting center.

Cases were chosen to reflect the heterogeneity of scenarios that may be faced in everyday practice. Particularly, three cases (cases 1, 7, and 10) were characterized by advanced donor age (>75 years). Of these, one (case 1) was further complicated by the association of significant graft macrovesicular steatosis (20%), whereas another one was characterized by graft allocation to an unstable recipient (case 7). In two cases (case 2 and 6), graft appeared steatotic, with a liver biopsy showing 40% macrovesicular steatosis (case 2). There were three cases of donation after circulatory death (DCD), one controlled (Maastricht 3, case 4) and two uncontrolled (Maastricht 2, case 3 and 9), all had prolonged warm ischemia time and underwent normothermic regional perfusion prior to retrieval. A pediatric donor with liver enzymes suggesting acute liver injury was also included (case 8). Two cases were classified as low complexity cases (case 5 and 6), whereas all others were classified as high‐complexity cases.

For each case, respondents were asked whether they would accept the offer (Q1), whether they would use MP (Q2) and, if so, which modality they would choose (Q3) and at what timing during preservation (Q4). A free comment could be added at the end of each case (Q5).

MP techniques were defined according to standard definitions.^26^ A sequential approach was defined as hypothermic MP followed by normothermic MP, with or without interposing a phase of controlled oxygenated rewarming.^17^

To investigate the perception of advantages and limitations of each different technique, contributors were specified to answer based not strictly on their center practice but on what they would have done in an ideal setting in which all techniques were available, free from any funding/logistic/resource restriction.

Data are expressed as number (%) and median (interquartile range) and compared using Mann‐Whitney, Chi‐square and Fisher tests, as appropriate. Interrater reliability was expressed using percentage of agreement and Krippendorff's alpha coefficient, which takes into account casual agreement by chance. Values vary from 1 (perfect agreement) to −1 (perfect disagreement, exceeding what can be expected by chance). A value of 0 reflects no interrater agreement beyond casual agreement by chance. Alpha > 0.8 is generally accepted as a measure of good interrater agreement. Statistical analyses and data visualization were performed using R: A language and environment for statistical computing (R Foundation for Statistical Computing, Vienna, Austria).

## 3 RESULTS

Response rate was 30.1% (39/127). Of respondents, 10 were senior figures or program directors at their Institution. Most respondents were from Europe (n = 35, 89.7%), whereas 3 (7.7%) and 1 (2.6%) were from North America and South America, respectively. Centre volume was ≥100, 50‐100, and <50 for 10 (25.6%), 19 (48.7%), and 10 (25.6%) respondents, respectively. Five respondents were from centers without an established MP program.

**FIGURE 1:**
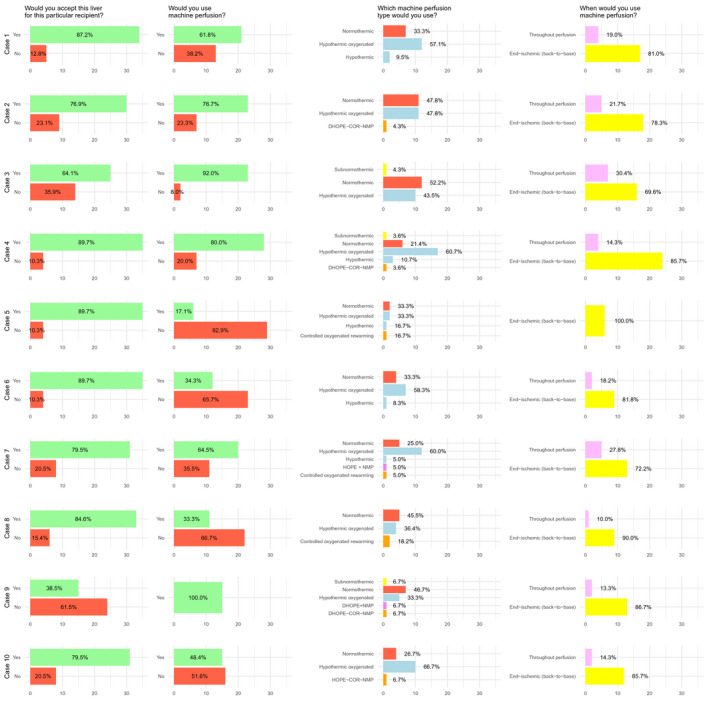
Survey answers [Color figure can be viewed at wileyonlinelibrary.com]

**FIGURE 2:**
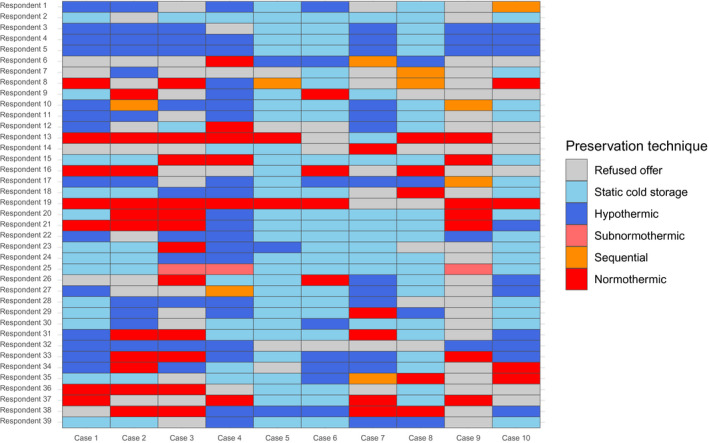
Heatmap depicting respondents’ attitude with regard to graft acceptance and choice of preservation method. Sequential approach includes cases in which hypothermic machine perfusion (MP) was followed by normothermic MP, with or without interposing a phase of controlled oxygenated rewarming. Resp, respondent (followed by sequential number) [Color figure can be viewed at wileyonlinelibrary.com]

Figures 1 and 2 summarize participants’ choices in each case, highlighting significant heterogeneity toward MP indication, modality, and graft acceptance. Lack of agreement between participants was confirmed by interrater reliability analysis, which showed Krippendorff's alpha coefficient never exceeding 0.15 for any of the survey questions. Lack of agreement persisted even when specific subsets (elderly donors, steatotic grafts, DCD donors, high‐complexity and low‐complexity cases) were analyzed separately (Table 2).

**TABLE 2:** Agreement between respondents according to setting and complexity of cases

		All cases	Age > 75	Steatotic	DCD	Low complexity	High complexity
Q1: graft acceptance							
PA		69%	70%	73%	62%	77%	68%
*α*		0.11	−0.01	0.02	0.17	−0.01	0.12
CI		(−0.05, 0.27)	(−0.03, 0.02)	(−0.10, 0.14)	(−0.27, 0.62)	(−0.02, 0.01)	(−0.05, 0.29)
Q2: use of MP (yes/no)							
PA		45%	38%	43%	51%	50%	44%
*α*		0.14	−0.01	0.09	0.11	0.01	0.09
CI		(0.04, 0.25)	(−0.02, 0.01)	(0.07, 0.12)	(−0.15, 0.37)	(−0.04, 0.07)	(−0.01, 0.20)
Q3: MP preferred approach							
PA		33%	26%	32%	34%	46%	30%
*α*		0.11	−0.01	0.06	0.09	0.01	0.08
CI		(0.02, 0.20)	(−0.02, 0.01)	(−0.05, 0.18)	(−0.11, 0.30)	(−0.04, 0.05)	(−0.01, 0.16)
Q4: Timing of MP							
PA		38%	30%	37%	41%	50%	35%
*α*		0.12	−0.01	0.08	0.09	0.01	0.08
CI		(0.02, 0.21)	(−0.02, 0.01)	(−0.05, 0.21)	(−0.12, 0.30)	(−0.03, 0.042)	(−0.01, 0.17)

Notes

Subgroups: Age > 75, case 1, 7 and 10; Steatotic, case 2 and 6; DCD, case 3, 4 and 9; Low complexity, case 5 and 6; High complexity, case 1, 2, 3, 4, 7, 8, 9, 10.

Abbreviations: CI, confidence interval for Krippendorff's alpha coefficient; DCD, donation after circulatory death; MP, machine perfusion; PA, percentage of agreement; Q, question; *α*, Krippendorff's alpha coefficient.

Of 390 theoretical maximum times that MP could have been deemed indicated (ie, in case all respondents would have used MP in all survey cases), MP was deemed indicated 174 times (44.6%). Hypothermic MP was the most preferred approach in 98 (56.3%) cases, followed by normothermic (n = 63, 36.2%), sequential (n = 10, 5.7%), and subnormothermic MP (n = 3, 1.7%). One participant (2.6%) did not consider MP useful in any case, whereas 18 (46.2%), 17 (43.6%), and 3 (7.7%) considered one, two, or three different MP approaches, respectively. Preference for an MP technique varied significantly according to the presence of an established MP program, with participants from centers without an MP program favoring more frequently hypothermic MP (Table 3).

**TABLE 3:** Preferred machine perfusion approach

n	Overall	MP program		*P* *
		No	Yes	
	174	19	155	
MP technique				<.01
Hypothermic	98 (56.3)	12 (63.2)	86 (55.5)	
Normothermic	63 (36.2)	3 (15.8)	60 (38.7)	
Sequential approach	10 (5.7)	1 (5.3)	9 (5.8)	
Subnormothermic	3 (1.7)	3 (15.8)	0 (0.0)	

Note

Data are presented as number (%).

* Chi‐square test comparing group with and without an established MP program.

When MP was indicated, an end‐ischemic approach was preferred in 137 (81.1%) cases, whereas continuous perfusion throughout preservation was chosen in 32 (18.9%). In five cases, preferred timing was not indicated. The choice of the technique influenced timing, with hypothermic perfusion being associated more frequently with an end‐ischemic approach and normothermic perfusion with continuous use throughout preservation (Table 4).

**TABLE 4:** Timing of machine perfusion according to technique

	End‐ischemic (back‐to‐base)	Throughout perfusion	*P* **
Hypothermic	84 (61.3)	11 (34.4)	<.01
Normothermic	44 (32.1)	17 (53.1)	
Sequential*	9 (6.6)	1 (3.1)	
Subnormothermic	0 (0.0)	3 (9.4)	

Note

Data are expressed as number (%) and represent the aggregate number of times each timing (end‐ischemic versus throughout perfusion) was chosen according to MP technique.

* Sequential approach includes cases in which hypothermic MP was followed by normothermic MP, with or without interposing a phase of controlled oxygenated rewarming

** Chi‐square test.

Finally, of 38 free comments to survey cases, 17 (44.7%) stressed the need for viability testing before transplant in cases 1, 2, 3, 4, 6, 9, and 10. Viability testing invariably involved the use of normothermic MP, either alone or in the setting of a sequential approach. Other comments were about logistic/organizational aspects (n = 10), the need of ruling out significant atherosclerosis of the hepatic artery (n = 3), the utility of obtaining a liver biopsy (n = 2), or other issues (n = 6). A full list of participants comments is available in Table S1.

## 4 DISCUSSION

The results of this survey could be summarized in three main points. First, there were significant discrepancies in MP indications and technique among participants and, more surprisingly, the same heterogeneity was observed with regard to graft acceptance, which was equally highly variable. Second, while approximately half of the participants stuck to one favorite technique regardless of the peculiarities of each case, the other half proposed two or more different approaches to be adapted to different scenarios. Third, the majority of comments stressed viability assessment as a fundamental aspect of MP, which was invariably linked to the use of normothermic MP.

MP indications and techniques are strongly varying, which can be attributed to the very recent clinical reintroduction of this technology after the early experiences in the pioneering era of solid organ transplantation.^27^ As of today, three randomized controlled trials have been published,^9^, ^11^, ^19^ one of which after the completion of this survey.^19^ Along with other retrospective studies, current literature suggests that MP conveys a significant advantage over static cold storage in several settings, including DCD donors,^7^, ^15^, ^18^, ^19^ extended‐criteria DBD donors,^12^, ^14^ elderly donors^9^ and steatotic grafts.^10^ However, the thresholds and clinical criteria for its application are difficult to determine in the real‐life practice, all the more that use of MP, at least initially, somewhat alters standard retrieval‐transplant routine and increases the costs of allograft preservation.^28^ In this context, recent guidelines proposed by the Italian Society for Organ Transplantation (SITO)^29^ and the International Liver Transplantation Society (ILTS)^30^, ^31^ represent a valuable effort to help clinicians evaluating MP indication in different settings and designing future trials on liver MP.

As expected, the choice of MP technique was arbitrary, subjective, or center‐related. Indeed, early studies have assessed feasibility of MP in a clinical setting or compared MP with SCS. No study comparing different MP techniques head‐to‐head has been published so far.^28^ However, results of a large, multicenter study comparing end‐ischemic normothermic versus end‐ischemic hypothermic oxygenated MP from Germany are expected and will hopefully shed some light on the respective benefits and shortcomings of each technique.

In this survey, hypothermic MP was chosen in 56.3% of cases, and an end‐ischemic approach was by far the most preferred timing (81.1% of cases). The reason why most respondents tended to prefer hypothermic MP might be at least partially related to the fact that hypothermic MP is frequently perceived as technically easier to perform and associated with inferior technology‐related risks. Noteworthy, results of the DHOPE‐DCD trial,^19^ favoring the use of hypothermic MP over static cold storage, were published after the completion of this survey.

Concerning MP timing, the vast preference toward an end‐ischemic approach likely reflects the perceived logistical challenges linked to backtable preparation and MP setup at the retrieval hospital and the risks associated with potential device malfunction during organ transport. However, it is worth noticing that current evidence^11^, ^32^ does not support the concept that preservation by upfront MP is associated with increased risk.

An interesting and rather unexpected finding was that half of the respondents proposed more than one technique to be applied in different scenarios. As any MP technique requires a certain degree of expertise, we would have expected that most respondents would feel more comfortable with one particular technique. In contrast, half of the respondents chose at least two different techniques for the proposed scenarios, suggesting that different techniques were perceived as associated with distinct characteristics and goals. Some respondents considered some techniques not as competitive, but rather as complementary, such as end‐ischemic hypothermic MP for initial resuscitation of the graft, followed by controlled oxygenated rewarming and subsequent normothermic MP for viability assessment.^17^

Finally, viability assessment was frequently indicated as a unique possibility offered by MP. In this regard, MP has radically changed the concept of organ preservation, creating a time window during which a functioning organ can be inspected and evaluated ex situ. This concept has already allowed successfully recovering and transplanting organs previously deemed unsuitable for LT.^17^, ^20^, ^33^ In this survey, viability assessment were invariably associated with the use of normothermic MP. However, as brilliantly summarized by Brüggenwirth et al,^34^, ^35^ viability assessment is still an imperfect science, as many criteria have been proposed but none validated. Many criteria focus on hepatocyte function, whereas cholangiocyte function and injury have been rather neglected.^2^, ^36^ Furthermore, some promising findings from the Zurich group suggest that viability assessment is possible also during hypothermic MP, although validation of this claim is still lacking.^37^ Ultimately, viability assessment appears to be a fundamental need of the clinician toward both hypothermic and normothermic MP technologies, especially in cases characterized by significant uncertainty regarding the suitability and function of the allograft.

Taken together, these findings depict a picture of nonuniform MP practice among experts in the field and highlight the urgent need for more clinical data and studies to make an evidence‐based approach to MP utilization possible. The recently released ILTS consensus guidelines on ex situ liver MP have addressed relevant aspects and highlighted potential pitfalls of future MP trials, tracing the path toward a more solid evidence in the field.^30^, ^31^ As proposed, a necessary step in this direction would be wide and transparent data sharing by creation of registries detailing indications and outcome of MP cases. Future studies should focus on clinically relevant endpoints, whereas recently introduced scores^38^, ^39^, ^40^ could be used as measures of post‐LT graft function.

This study has limitations, including limited sample size (n = 39) and response rate (30.1%), although this last was in line with that of other web‐based medical surveys.^41^ As the majority of participants were from Europe, results do not accurately reflect practices elsewhere. Significant differences in donation rate, waiting list time, surgeons personal experience and country‐specific protocols, especially with regards to DCD donors (systematic use of end‐ischemic hypothermic MP vs. normothermic regional perfusion), could have influenced variance in answers. As an example, use of normothermic regional perfusion in the proposed DCD cases was obligatory at the promoting center, and this is why this was not considered as a further preservation option. However, normothermic regional perfusion undoubtedly represents another variable in the equation and its use, alone or in association with MP, certainly deserves evaluation.^8^, ^42^ One strength of this survey was its being targeted to people with direct hands‐on experience with MP and with a solid background about the advantages and limitations of each technique.

In conclusion, just like what happens when evaluating an organ offer, it appears that the choice of applying MP is based on an important element of subjectivity and regional differences.^43^ Every clinician likely takes into account several donor, recipient and logistic factors, which are weighed based on her/his experience, retrieval organization and waitlist pressure. This highlights the need for high‐quality studies, focusing on clearly defined settings and having strong clinical endpoints, to drive clinical MP use in everyday practice.

## CONFLICT OF INTEREST

The authors declare that they have no conflicts of interest with the contents of this article.

## AUTHORS’ CONTRIBUTIONS

DP: concept and design, data collection, analysis and interpretation, and drafting the article; DC, FR: concept and design, and data collection; RA, MIB, EB‐R, IMAB, ZC, RDC, VEDM, DD, DE, DG, AJH, DK, QL, GL, TMM, AM, FM, DN, AN, DP, MR, MCS, AW, PDS, CF, WJ, MM, RJP, MR, MS, MS: data interpretation, critical revision of article, and approval of the article; AWA: concept and design, data interpretation, critical revision of article, and approval of article; RR: data interpretation, critical revision of article, and approval of the article.

## Supporting information

Supplementary Material

## APPENDIX A

**The Liver Machine Perfusion Survey Group**: Roberta Angelico (HPB and Transplant Unit, Department of Surgery Science, Tor Vergata University, Rome, Italy); Maria Irene Bellini (Department of Emergency Medicine and Surgery, Azienda Ospedaliera San Camillo Forlanini, Rome, Italy; Department of Surgical Sciences, Sapienza Università di Roma, Rome, Italy); Eliano Bonaccorsi‐Riani (Starzl Unit of Abdominal Transplantation, Cliniques Universitaires Saint‐Luc, Université catholique de Louvain, Brussels, Belgium; Pôle de Chirurgie Expérimentale et Transplantation, Université Catholique de Louvain, Brussels, Belgium); Isabel M. A. Brüggenwirth (Department of Surgery, University of Groningen, University Medical Center Groningen, Groningen, The Netherlands); Zoltan Czigany (Department of Surgery and Transplantation, University Hospital RWTH Aachen, Aachen, Germany); Riccardo De Carlis (Department of General Surgery and Transplantation, ASST Grande Ospedale Metropolitano Niguarda, Milan, Italy); Vincent E. De Meijer (Department of Surgery, University of Groningen, University Medical Center Groningen, Groningen, The Netherlands); Daniele Dondossola (General and Liver Transplant Surgery Unit, Fondazione IRCCS Ca' Granda Ospedale Maggiore Policlinico, Milan, Italy); Dilmurodjon Eshmuminov (Department of Surgery and Transplantation, Swiss Hepato‐Pancreato‐Biliary (HPB) Center, University Hospital Zurich, Zurich, Switzerland); Davide Ghinolfi (Division of Hepatic Surgery and Liver transplantation, University Hospital of Pisa, Pisa, Italy); Amelia J. Hessheimer (Hepatopancreatobiliary Surgery & Transplantation, Hospital Clínic Barcelona, IDIBAPS, CIBERehd, University of Barcelona, Barcelona, Spain); Dagmar Kollmann (Department of Surgery, Division of Transplantation, Medical University of Vienna, Vienna, Austria ); Quirino Lai (Hepato‐biliary and Organ Transplant Unit, Department of Surgery, Sapienza University of Rome, Viale del Policlinico 155, 00161 Rome, Italy); Georg Lurje (Department of Surgery, Campus Charité Mitte, Campus Virchow‐Klinikum, Charité‐Universitätsmedizin Berlin, Berlin, Germany); Tommaso M. Manzia (HPB and Transplant Unit, Department of Surgery Science, Tor Vergata University, Rome, Italy); Arianeb Merhabi (Department of General, Abdominal and Transplant Surgery, Ruprecht‐Karls University of Heidelberg, Heidelberg, Germany); Fabio Melandro (Division of Hepatic Surgery and Liver transplantation, University Hospital of Pisa, Pisa, Italy); David Nasralla (Department of HPB & Liver Transplant Surgery, Royal Free Hospital, London, UK); Arash Nickkholgh (Department of General, Abdominal and Transplant Surgery, Ruprecht‐Karls University of Heidelberg, Heidelberg, Germany); Duilio Pagano (Department for the Treatment and Study of Abdominal Diseases and Abdominal Transplantation, IRCCS‐ISMETT, UPMC Italy, Palermo, Italy); Michel Rayar (CHU Rennes, Service de Chirurgie Hépatobiliaire et Digestive, F‐35033 Rennes, France); Maria Cristina Saffioti (Division of Hepatobiliopancreatic Surgery, Liver and Kidney Transplantation, Research Unit of Clinical Hepatogastroenterology and Transplantation, Bambino Gesù Children's Hospital, IRCCS, Piazza Sant'Onofrio, 4, Rome, Italy); Annemarie Weissenbacher (Department of Visceral, Transplant and Thoracic Surgery, Medical University of Innsbruck, Innsbruck, Austria); Alfonso W. Avolio (General Surgery and Liver Transplantation Unit, Fondazione Policlinico Universitario Agostino Gemelli, IRCCS, Rome, Italy); Paolo De Simone (Division of Hepatic Surgery and Liver transplantation, University Hospital of Pisa, Pisa, Italy); Costantino Fondevila (Hepatopancreatobiliary Surgery & Transplantation, Hospital Clínic Barcelona, IDIBAPS, CIBERehd, University of Barcelona, Barcelona, Spain); Wayel Jassem (Institute of Liver Studies, King’s College Hospital, SE5 9RS London, UK); Malcolm Macconmara (Surgery, Solid Organ Transplant Program, UT Southwestern Medical Center, Dallas, TX, USA); Robert J. Porte (Department of Surgery, University of Groningen, University Medical Center Groningen, Groningen, The Netherlands); Matteo Ravaioli (Department of Medical and Surgical Sciences (DIMEC), University of Bologna, Bologna, Italy; Department of General Surgery and Transplantation, IRCCS, Azienda Ospedaliero‐Universitaria di Bologna, Bologna, Italy); Markus Selzner (Multi Organ Transplant Program, Department of Surgery, Toronto General Hospital, Canada); Marco Spada (Division of Hepatobiliopancreatic Surgery, Liver and Kidney Transplantation, Research Unit of Clinical Hepatogastroenterology and Transplantation, Bambino Gesù Children's Hospital, IRCCS, Piazza Sant'Onofrio, 4, Rome, Italy).

## References

[1] Friend PJ , Ploeg RJ . One‐week perfusion of human livers: how far will we go? Transplantation. 2020;104:1756–7. 32826838 10.1097/TP.0000000000003286

[2] Eshmuminov D , Becker D , Bautista Borrego L , Hefti M , Schuler MJ , Hagedorn C , et al. An integrated perfusion machine preserves injured human livers for 1 week. Nat Biotechnol. 2020;38:189–98. 31932726 10.1038/s41587-019-0374-x PMC7008032

[3] Bral M , Dajani K , Leon Izquierdo D , Bigam D , Kneteman N , Ceresa CDL , et al. A back‐to‐base experience of human normothermic ex situ liver perfusion: does the chill kill? Liver Transpl. 2019;25:848–58. 30938039 10.1002/lt.25464

[4] Cardini B , Oberhuber R , Fodor M , Hautz T , Margreiter C , Resch T , et al. Clinical implementation of prolonged liver preservation and monitoring through normothermic machine perfusion in liver transplantation. Transplantation. 2020;104:1917–28. 32371845 10.1097/TP.0000000000003296

[5] Ceresa CDL , Nasralla D , Watson CJE , Butler AJ , Coussios CC , Crick K , et al. Transient cold storage prior to normothermic liver perfusion may facilitate adoption of a novel technology. Liver Transpl. 2019;25:1503–13. 31206217 10.1002/lt.25584

[6] Cussa D , Patrono D , Catalano G , Rizza G , Catalano S , Gambella A , et al. Use of dual hypothermic oxygenated machine perfusion to recover extended criteria pediatric liver grafts. Liver Transpl. 2020;26:835–9. 32196915 10.1002/lt.25759

[7] De Carlis R , Schlegel A , Frassoni S , Olivieri T , Ravaioli M , Camagni S , et al. How to Preserve liver grafts from circulatory death with long warm ischemia? A retrospective Italian cohort study with normothermic regional perfusion and hypothermic oxygenated perfusion. Transplantation. 2021. 10.1097/TP.0000000000003595 33617211

[8] Ghinolfi D , Dondossola D , Rreka E , Lonati C , Pezzati D , Cacciatoinsilla A , et al. Sequential use of normothermic regional and ex situ machine perfusion in donation after circulatory death liver transplant. Liver Transpl. 2021;27:385–402. 10.1002/lt.25899 32949117

[9] Ghinolfi D , Rreka E , De Tata V , Franzini M , Pezzati D , Fierabracci V , et al. Pilot, open, randomized, prospective trial for normothermic machine perfusion evaluation in liver transplantation from older donors. Liver Transpl. 2019;25:436–49. 10.1002/lt.25362 30362649

[10] Kron P , Schlegel A , Mancina L , Clavien PA , Dutkowski P . Hypothermic oxygenated perfusion (HOPE) for fatty liver grafts in rats and humans. J Hepatol. 2017:S0168‐8278(17)32268‐7. 10.1016/j.jhep.2017.08.028 28870676

[11] Nasralla D , Coussios CC , Mergental H , Akhtar MZ , Butler AJ , Ceresa CDL , et al. A randomized trial of normothermic preservation in liver transplantation. Nature, 2018;557:50–6. 29670285 10.1038/s41586-018-0047-9

[12] Patrono D , Surra A , Catalano G , Rizza G , Berchialla P , Martini S , et al. Hypothermic oxygenated machine perfusion of liver grafts from brain‐dead donors. Sci Rep. 2019;9:9337. 31249370 10.1038/s41598-019-45843-3 PMC6597580

[13] Ravaioli M , De Pace V , Angeletti A , Comai G , Vasuri F , Baldassarre M , et al. Hypothermic oxygenated new machine perfusion system in liver and kidney transplantation of extended criteria donors: first Italian clinical trial. Sci Rep. 2020;10:6063. 32269237 10.1038/s41598-020-62979-9 PMC7142134

[14] Rayar M , Maillot B , Bergeat D , Camus C , Houssel‐Debry P , Sulpice L , et al. A preliminary clinical experience using hypothermic oxygenated machine perfusion for rapid recovery of octogenarian liver grafts. Prog Transplant. 2019;29:97–8. 10.1177/1526924818817072 30497334

[15] Schlegel A , Muller X , Kalisvaart M , Muellhaupt B , Perera MTPR , Isaac JR , et al. Outcomes of DCD liver transplantation using organs treated by hypothermic oxygenated perfusion before implantation. J Hepatol. 2019;70:50–7. 10.1016/j.jhep.2018.10.005 30342115

[16] Selzner M , Goldaracena N , Echeverri J , Kaths JM , Linares I , Selzner N , et al. Normothermic ex vivo liver perfusion using steen solution as perfusate for human liver transplantation: first North American results. Liver Transpl. 2016;22:1501–8. 27339754 10.1002/lt.24499

[17] van Leeuwen OB , de Vries Y , Fujiyoshi M , Nijsten MWN , Ubbink R , Pelgrim GJ , et al. Transplantation of high‐risk donor livers after ex situ resuscitation and assessment using combined hypo‐ and normothermic machine perfusion: a prospective clinical trial. Ann Surg 2019;270:906–14. 31633615 10.1097/SLA.0000000000003540

[18] van Rijn R , Karimian N , Matton APM , Burlage LC , Westerkamp AC , van den Berg AP , et al. Dual hypothermic oxygenated machine perfusion in liver transplants donated after circulatory death. Br J Surg. 2017;104:907–17. 28394402 10.1002/bjs.10515 PMC5484999

[19] van Rijn R , Schurink IJ , de Vries Y , van den Berg AP , Cortes Cerisuelo M , Darwish Murad S , et al. Hypothermic machine perfusion in liver transplantation—a randomized trial. N Engl J Med. 2021;384:1391–401. 33626248 10.1056/NEJMoa2031532

[20] Watson CJE , Kosmoliaptsis V , Pley C , Randle L , Fear C , Crick K , et al. Observations on the ex situ perfusion of livers for transplantation. Am J Transplant. 2018;18:2005–20. 29419931 10.1111/ajt.14687 PMC6099221

[21] Rayar M , Beaurepaire J‐M , Bajeux E , Hamonic S , Renard T , Locher C , et al. Hypothermic oxygenated perfusion improves extended criteria donor liver graft function and reduces duration of hospitalization without extra cost: the PERPHO study. Liver Transpl. 2021;27:349–62. 33237618 10.1002/lt.25955

[22] Patrono D , Catalano G , Rizza G , Lavorato N , Berchialla P , Gambella A , et al. Perfusate analysis during dual hypothermic oxygenated machine perfusion of liver grafts: correlations with donor factors and early outcomes. Transplantation. 2020;104:1929–42. 32769628 10.1097/TP.0000000000003398

[23] Patrono D , Lavezzo B , Molinaro L , Rizza G , Catalano G , Gonella F , et al. Hypothermic oxygenated machine perfusion for liver transplantation: an initial experience. Exp Clin Transplant. 2018;16:172–6. 29108514 10.6002/ect.2016.0347

[24] Czigany Z , Pratschke J , Fronek J , Guba M , Schoning W , Raptis DA , et al. Hypothermic oxygenated machine perfusion reduces early allograft injury and improves post‐transplant outcomes in extended criteria donation liver transplantation from donation after brain death. Ann Surg. 2021;274. 10.1097/SLA.0000000000005110 34334635

[25] Ignatavicius P , Oberkofler CE , Chapman WC , DeMatteo RP , Clary BM , D’Angelica MI , et al. Choices of therapeutic strategies for colorectal liver metastases among expert liver surgeons: a throw of the dice? Ann Surg. 2020;272:715–22. 32833764 10.1097/SLA.0000000000004331

[26] Burra P , Zanetto A , Russo FP , Germani G . Organ preservation in liver transplantation. Semin Liver Dis. 2018;38:260–9. 30041278 10.1055/s-0038-1666840

[27] Belzer FO , Ashby BS , Dunphy JE . 24‐hour and 72‐hour preservation of canine kidneys. Lancet. 1967;2:536–8. 4166894 10.1016/s0140-6736(67)90498-9

[28] Bonaccorsi‐Riani E , Bruggenwirth IMA , Buchwald JE , Iesari S , Martins PN . Machine perfusion: cold versus warm, versus neither. update on clinical trials. Semin Liver Dis. 2020;40:264–81. 32557478 10.1055/s-0040-1713118

[29] Ghinolfi D , Lai Q , Dondossola D , De Carlis R , Zanierato M , Patrono D , et al. Machine perfusions in liver transplantation: the evidence‐based position paper of the Italian society of organ and tissue transplantation. Liver Transpl. 2020;26:1298–315. 32519459 10.1002/lt.25817

[30] Martins PN , Rizzari MD , Ghinolfi D , Jochmans I , Attia M , Jalan R , et al. Design, analysis, and pitfalls of clinical trials using ex situ liver machine perfusion: the international liver transplantation society consensus guidelines. Transplantation. 2021;105:796–815. 33760791 10.1097/TP.0000000000003573

[31] Quintini C , Muiesan P , Detry O , Gastaca M , de Jonge J , Clavien P‐A , et al. Early allograft dysfunction and complications in DCD liver transplantation: expert consensus statements from the international liver transplantation society. Transplantation. 2021;105:1643–52. 34291765 10.1097/TP.0000000000003877

[32] Ravikumar R , Jassem W , Mergental H , Heaton N , Mirza D , Perera MT , et al. Liver transplantation after ex vivo normothermic machine preservation: a phase 1 (first‐in‐man) clinical trial. Am J Transplant. 2016;16:1779–87. 26752191 10.1111/ajt.13708

[33] Mergental H , Laing RW , Kirkham AJ , Perera MTPR , Boteon YL , Attard J , et al. Transplantation of discarded livers following viability testing with normothermic machine perfusion. Nat Commun. 2020;11:2939. 32546694 10.1038/s41467-020-16251-3 PMC7298000

[34] Bruggenwirth IMA , de Meijer VE , Porte RJ , Martins PN . Viability criteria assessment during liver machine perfusion. Nat Biotechnol. 2020;38:1260–2. 33106683 10.1038/s41587-020-0720-z

[35] Bruggenwirth IMA , van Leeuwen OB , Porte RJ , Martins PN . The emerging role of viability testing during liver machine perfusion. Liver Transpl. 2021. 10.1002/lt.26092 33963657

[36] Matton APM , de Vries Y , Burlage LC , van Rijn R , Fujiyoshi M , de Meijer VE , et al. Biliary bicarbonate, pH, and glucose are suitable biomarkers of biliary viability during ex situ normothermic machine perfusion of human donor livers. Transplantation. 2019;103:1405–13. 30395120 10.1097/TP.0000000000002500 PMC6613725

[37] Muller X , Schlegel A , Kron P , Eshmuminov D , Würdinger M , Meierhofer D , et al. Novel real‐time prediction of liver graft function during hypothermic oxygenated machine perfusion before liver transplantation. Ann Surg. 2019;270:783–90. 31592808 10.1097/SLA.0000000000003513

[38] Agopian VG , Harlander‐Locke MP , Markovic D , Dumronggittigule W , Xia V , Kaldas FM , et al. Evaluation of early allograft function using the liver graft assessment following transplantation risk score model. JAMA Surg. 2018;153:436–44. 29261831 10.1001/jamasurg.2017.5040 PMC6584313

[39] Avolio AW , Franco A , Schlegel A , Lai Q , Meli S , Burra P , et al. Development and validation of a comprehensive model to estimate early allograft failure among patients requiring early liver retransplant. JAMA Surg. 2020;155:e204095. 33112390 10.1001/jamasurg.2020.4095 PMC7593884

[40] Avolio AW , Lai Q , Cillo U , Romagnoli R , De Simone P . L‐GrAFT and EASE scores in liver transplantation: need for reciprocal external validation and comparison with other scores. J Hepatol. 2021;75:729–31. 10.1016/j.jhep.2020.12.009 33340580

[41] Cunningham CT , Quan H , Hemmelgarn B , Noseworthy T , Beck CA , Dixon E , et al. Exploring physician specialist response rates to web‐based surveys. BMC Med Res Methodol. 2015;15:32. 25888346 10.1186/s12874-015-0016-z PMC4404667

[42] Hessheimer AJ , Coll E , Torres F , Ruíz P , Gastaca M , Rivas JI , et al. Normothermic regional perfusion vs. super‐rapid recovery in controlled donation after circulatory death liver transplantation. J Hepatol. 2019;70:658–65. 30582980 10.1016/j.jhep.2018.12.013

[43] Watson CJE , Jochmans I . From, “gut feeling” to objectivity: machine preservation of the liver as a tool to assess organ viability. Curr Transplant Rep. 2018;5:72–81. 29564205 10.1007/s40472-018-0178-9 PMC5843692

