# Supplementary material for: Heterogeneous indications and the need for viability assessment: An international survey on the use of machine perfusion in liver transplantation
Source: Artif Organs. 2021 Sep 8;46(2):296–305. doi: 10.1111/aor.14061 (PMC9291461; doi:10.1111/aor.14061)
Supplement: Supplementary file 1 — Supplementary Material [file AOR-46-296-s001.docx]

**Supplementary Material 1.** Full survey text

**Introduction**

In this brief survey, we would like to know your attitude concerning the use of machine perfusion in 10 scenarios of donor recipient matching in liver transplantation. Answers should reflect what you WOULD DO (provided you have all technology available and no funding/logistic restrictions) and not necessarily what you regularly do in your everyday practice.

The aim of this survey is investigating indications for machine perfusion in liver transplantation across different transplant centres.

Collected data will be treated anonymously and in an aggregated fashion (Reg. EU 2016/679).

If you wish, at the end of the survey you will be able to know how these cases were actually managed.

Thank you for your time and enjoy the survey!

The Promoting Centre Team

**Case 1**

Donor features:

- DBD

- Age = 78

- BMI = 32

- Expected ischemia time = 11 hours

- Liver enzymes: AST 41 IU/L; ALT 39 IU/L

- Inotropes: norepinephrine (stable haemodynamics)

- Evaluation by retrieving surgeon = marked steatosis, round margins, dysmorphic

- Biopsy available = Yes (macrovesicular steatosis = 20%)

Recipient features:

- Indication = HCC on NASH cirrhosis

- Age = 66

- BMI = 27

- MELD = 10

- Status = HOME

- Urgency = ELECTIVE

Management:

With the aim of assessing liver viability and minimising ischemia-reperfusion injury, normothermic machine perfusion was used for 309 minutes, during which liver was deemed transplantable. Graft function was good, with AST and ALT peak of 1200 IU/L and 883 IU/l, respectively. Postoperative course was uneventful. Patient died with a functioning graft and no evidence of ischemic cholagiopathy 6 months after transplant due to HCC recurrence.

**Case 2**

Donor features:

- DBD

- Age = 63

- BMI = 27

- Expected ischemia time = 6 hours

- Liver enzymes: AST 67 IU/L; ALT 81 IU/L

- Inotropes: norepinephrine (stable)

- Evaluation by retrieving surgeon = marked steatosis

- Biopsy available =yes (macrovesicular steatosis = 40%)

Recipient features:

- Indication = HCC on NASH cirrhosis

- Age = 53

- BMI = 33

- MELD = 20

- Status = HOME

- Urgency = ELECTIVE

Management:

To reduce ischemia-reperfusion injury, end-ischemic D-HOPE for 120 minutes was used in this case. Preservation injury at time-zero biopsy was severe and transaminase peak was high (AST = 12,075; ALT = 8,835). Patient course was complicated by bleeding, which required relaparotomy, and stage 3 acute kidney injury, requiring dialysis. He developed delayed non-function and underwent retransplantation on postoperative day 31st. He made initially a good recovery but died 70 days after retransplant due to HHV8 infection.

**Case 3**

Donor features:

- DCD (Maastricht cat. 2)

- Age = 49

- BMI = 28

- Expected ischemia time = 11 hours

- Inotropes: vasopressine

- Evaluation by retrieving surgeon = normal parenchyma

- Biopsy available = yes (macrovesicular steatosis = 10%)

- Normothermic regional perfusion was started after a low-flow time of about 120 minutes and 35 minutes of asystolic warm ischemia time.

- Normothermic regional perfusion lasted 278 minutes, during which maximum lactate fell from 17.9 to 9.1 mmol/L, AST increased from 230 to 341 IU/L and ALT increased from 63 to 260 IU/L.

Recipient features:

- Indication = NASH cirrhosis

- Age = 59

- BMI = 22

- MELD = 21

- Status = HOME

- Urgency = ELECTIVE

Management:

The offer was accepted and D-HOPE was used for 240 minutes. Time-zero biopsy showed mild reperfusion injury and transaminase peak was low (AST = 222 IU/L; ALT = 333 IU/l). Graft function was good. Postoperative course was complicated by acute cellular rejection (RAI 4/9) and Tacrolimus neurotoxicity. Patient is alive 12 month after transplant with no evidence of ischemic cholangiopathy.

**Case 4**

Donor features:

- DCD (Maastricht cat. 3)

- Age = 56

- BMI = 24

- Expected ischemia time = 8 hours

- Inotropes: NO

- Evaluation by retrieving surgeon = normal

- Biopsy available = YES (macrovesicular steatosis = 5%)

- Functional warm ischemia time was 49 minutes; normothermic regional perfusion was used for 198 minutes, during which lactate fell from 11.5 to 1.9 mmol/L, AST increased from 135 to 200 IU/L and ALT increased from 117 to 131 IU/L

Recipient features:

- Indication: HCC on alcoholic cirrhosis

- Age = 54

- BMI = 22

- MELD = 10

- Status = HOME

- Urgency = ELECTIVE

Management:

End-ischemic D-HOPE during 135 minutes was used in this case. Preservation injury at time-zero biopsy was mild and AST/ALT peak was 1024 IU/L and 596 IU/L, respectively. Graft and patient made a good recovery, with postoperative course being characterised by postoperative delirium. Patient is alive 10 months after transplant with no evidence of ischemic cholangiopathy.

**Case 5**

Donor features:

- DBD

- Age = 52

- BMI = 25

- Expected ischemia time = 6,5 hours

- Liver enzymes: AST 170 IU/L; ALT 216 IU/L

- Inotropes: NO

- Evaluation by retrieving surgeon = transplantable

- Biopsy available = NO

Recipient features:

- Indication = HCC on alcoholic cirrhosis

- Age = 52

- BMI = 28

- MELD = 7

- Status = HOME

- Urgency = ELECTIVE

Management:

End-ischemic D-HOPE was used for 100 minutes. AST and ALT peak was 872 IU/L and 879 IU/L, respectively. Graft function was excellent and patient was discharged home on postoperative day 7th. Patient is alive 14 months after transplant with no evidence of ischemic cholangiopathy.

**Case 6**

Donor features:

- DBD

- Age = 42

- BMI = 31

- Expected ischemia time = 6 hours

- Liver enzymes: AST 32 IU/L; ALT 125 IU/L

- Inotropes: norepinephrine (stable hemodyanmics)

- Evaluation by retrieving surgeon = big liver, steatotic but transplantable

- Biopsy available = NO

Recipient features:

- Indication = HCC on HCV-related cirrhosis

- Age = 53

- BMI = 27

- MELD = 8

- Status = HOME

- Urgency = ELECTIVE

Management:

Graft (weighing 2.7 kg) was treated with end-ischemic D-HOPE for 157 minutes. Time-zero biopsy showed 20% macrosteatosis and mild preservation injury. Transaminase peak was elevated: AST = 5711 IU/L and ALT 2606 IU/L. Graft function eventually recovered after prolonged cholestasis, but patient required prolonged renal replacement therapy. 10 months after transplant he is alive and with no evidence of ischemic cholangiopathy.

**Case 7**

Donor features:

- DBD

- Age = 76

- BMI = 22

- Expected ischemia time = 10 hours

- Liver enzymes: AST 257 IU/L; ALT 304 IU/L

- Inotropes: high-dose norepinephrine (unstable hemodynamics)

- Evaluation by retrieving surgeon = normal parenchyma; vessels thickening and calcification

- Biopsy available = NO

Recipient features:

- Indication: Fulminant hepatitis (HBV-related)

- Age = 40

- BMI = 29

- MELD = 41

- Status = ICU

- Urgency = EMERGENCY

Management:

Normothermic machine perfusion (back-to base approach) was used for 307 minutes, with the primary aim of minimizing ischemia-reperfusion injury. Transplant operation was complicated by severely unstable hemodynamics and escalating inotrope requirement. Postoperatively, recipient lactate was > 20 mmol/L. Time-zero biopsy showed moderate preservation injury and no steatosis. Despite severe baseline condition, patient recovered quickly and graft exhibited excellent function (AST peak was 212 IU/L and ALT peak was 384 IU/l). At 20-month follow-up, patient is alive and well and no evidence of biliary complications.

**Case 8**

Donor features:

- Age = 12

- BMI = 15

- Expected ischemia time = 8 hours

- Liver enzymes: AST 680 IU/L; ALT 560 IU/L

- Inotropes: NO

- Evaluation by retrieving surgeon = normal parenchyma

- Biopsy available = NO

Recipient features:

- Indication = primary sclerosing cholangitis

- Age = 14

- BMI = 16

- MELD = 7

- Status = HOME

- Urgency = ELECTIVE

Management:

End-ischemic D-HOPE for 90 minutes was used given elevated donor transaminase. Time-zero biopsy showed mild reperfusion injury and no steatosis. AST and ALT peak was respectively 1121 IU/L and 1307 IU/L. Patient and graft made and excellent recovery. Mild acute cellular rejection was observed postoperatively. At 8-month follow-up, he presented with an isolated ischemic-type stricture of S6 duct, which was treated by percutaneous bilioplasty, and a tight stricture of the hepatico-jejunostomy, which was treated surgically by redo hepatico-jejunostomy. At 21-month follow-up he is alive and well and with normal liver function.

**Case 9**

Donor features:

- DCD (Maastricht cat. 2)

- Age = 21

- BMI = 28

- Expected ischemia time = 10 hours

- Baseline liver enzymes on arrival: AST 500 IU/L; ALT 600 IU/L

- Inotropes: norepinephrine (unstable hemodynamics)

- Evaluation by retrieving surgeon = accetable

- Biopsy available = YES (necrosis 25%)

- Normotermic regional perfusion was established after a period of 90-minutes low-flow and 31-minutes asystolic warm ischemia time. During NRP, lactate fluctuated around 9 mmol/L and transaminase level reached high levels (AST = 3500 IU/L; ALT = 1000 IU/L)

Recipient features:

- Indication = HCC on alcoholic cirrhosis

- Age = 56

- BMI = 26

- MELD = 9

- Status = HOME

- Urgency = ELECTIVE

Management:

Liver graft was treated by normothermic machine perfusion with the aim of assessing liver viability. Despite very quick lactate clearance of the machine, liver was discarded due to poor hepatic artery flow and inhomogeneous

perfusion.

**Case 10**

Donor features:

- DBD

- Age = 96

- BMI = 23

- Expected ischemia time = 9 hours

- Ezymes: AST 67 IU/L; ALT 95 IU/L

- Inotropes: norepinephrine (stable hemodynamics)

- Evaluation by retrieving surgeon = normal parenchyma

- Biopsy available: NO

Recipient features:

- Indication = HCC on HCV-related cirrhosis

- Age = 55

- BMI = 18

- MELD = 8

- Status = Home

- Urgency = Elective

Management:

End-ischemic D-HOPE for 120 minutes was used, mainly due to donor age. Time-zero biopsy showed moderate preservation injury and minimal steatosis. Transaminase peak was low (AST = 354 IU/L; ALT = 138 IU/L). Patient and graft made excellent recovery. Patient is alive and well 2.5 years after transplant, with no evidence of biliary complications.

| **Supplementary Table 1.** Comments according to type of MP | |
| --- | --- |
| Case 1 |  |
| Hypothermic oxygenated | I would have performed a liver biopsy |
| Normothermic | CIT 11hrs where primary for liver would not be realistic in most cases occuring in the US |
| Normothermic* | assess liver function ex vivo |
| Case 2 |  |
| Normothermic* | I would like to test the viability of the graft |
| Normothermic | RECIPIENT CHARATCTERISTICS MIGHT INFLUENCE INTRA- AND POST-OPERATIVE COURSES |
| Normothermic* | I would perform viability testing |
| DHOPE-COR-NMP* | Given the 40% steatosis this is likely a liver that will be declined by all centres in my country. The liver would then be offered for our combined DHOPE-COR-NMP protocol for resuscitation and viability testing. If it meets all of our vibility criteria, the liver would be transplanted |
| Normothermic | Would not accept post-crossclamp offer for this liver |
| Case 3 |  |
| Normothermic* | WE NEED TO EVALUATE THE LIVER PARENCHYMA FOR THE FIRST 1-2 HOURS OF EX-VIVO NMP. |
| Normothermic* | We would discard the liver if the lactate levels in the perfusate do not decrease. |
| Hypothermic oxygenated | two hours of end-ischemic DHOPE is standard practice in our center |
| Normothermic | I would use NMP at the donor hospital on worth, however, we have no funding for that at the moment |
| Normothermic* | Evaluation during normothermic perfusion - further decrease in lactate? bile production? |
| Normothermic | Unrealistic in US practice why a DCD should have expected 11 hours of cold ischemia. |
| Case 4 |  |
| Hypothermic | WE NEED TO EVALUATE A PROPER TIMING FOR REDUCING THE EFFECT COLD ISCHEMIA TIME |
| Hypothermic oxygenated | Although, in Germany no DCD is allowed, therefore only hypothetical scenario for our setting |
| DHOPE-COR-NMP* | to test viability and function |
| Hypothermic oxygenated | (D)HOPE preference for all DCD livers |
| Hypothermic oxygenated | End-ischemic DHOPE is standard practice for all DCD livers in our center |
| Normothermic* | NMP to assess organ performance |
| Case 6 |  |
| Hypothermic oxygenated | l would have performed a liver biopsy |
| Hypothermic oxygenated | Its optional I think to use MP in this scenario |
| Normothermic* | Only transplantable if the lactate concentration in the perfusate decrease throughout the perfusion |
| Normothermic* | I would perform viability testing on this liver |
| Hypothermic oxygenated | Although small doubt regarding function, some reconditioning before implantation would be beneficial |
| Case 7 |  |
| Hypothermic oxygenated | Only if atherosclerosis of common hepatic artery was absent |
| Hypothermic oxygenated | In this case we would machine perfuse due to the prolonged CIT. |
| HOPE + NMP | Would use considering an emergency, although it is necessary to evaluate the extension of arterial calcification because it may make the liver not transplantable. |
| Case 8 |  |
| Normothermic | My major concern is if the liver will overcome the extension of lesion |
| Normothermic | knowing the development of liver enzymes in the patient would be helpful |
| Controlled oxygenated rewarming | Is there an indication for transplantation for this recipient at all with this MELD? Are there other PSC associated criteria? |
| Case 9 |  |
| DHOPE+NMP* | NMP to evaluate the graft |
| Normothermic* | WE NEED TO EVALUATE LIVER PARENCHYMA PERFUSION DURING EX-VIVO NMP |
| DHOPE-COR-NMP* | This DCD liver has an increased risk for PNF and we would test this liver first, using our DHOPE-COR-NMP protocol |
| Normothermic* | To evaluate graft performance |
| Normothermic* | viability testing mandatory |
| Case 10 |  |
| HOPE-COR-NMP | No information on vessel status but if artery is good, yes |
| Normothermic* | Major concern is function |
| *comment about the need to perform viability testing before transplant. | |
